# A new enzymatic route for production of long 5'-phosphorylated oligonucleotides using suicide cassettes and rolling circle DNA synthesis

**DOI:** 10.1186/1472-6750-7-49

**Published:** 2007-08-16

**Authors:** Jakob S Lohmann, Magnus Stougaard, Jørn Koch

**Affiliations:** 1 Institute of Pathology, Aarhus University, 8000 Aarhus C, Denmark

## Abstract

**Background:**

The quality of chemically synthesized oligonucleotides falls with the length of the oligonucleotide, not least due to depurinations and premature termination during production. This limits the use of long oligonucleotides in assays where long high-quality oligonucleotides are needed (e.g. padlock probes). Another problem with chemically synthesized oligonucleotides is that secondary structures contained within an oligonucleotide reduce the efficiency of HPLC and/or PAGE purification. Additionally, ligation of chemically synthesized oligonucleotides is less efficient than the ligation of enzymatically produced DNA molecules.

**Results:**

Chemically synthesized oligonucleotides with hairpin structures were acquired from our standard supplier. The stem of the hairpin contained recognition sequences for the Nt. Alw I nicking enzyme and the Mly I restriction enzyme. These double stranded regions were positioned in a way to allow self-templated circularization of the oligonucleotide. Following ligation, tandem repeats of the complementary sequence of the circular oligonucleotide could be produced through rolling circle DNA synthesis. By running successive rounds of ligation, rolling circle DNA synthesis, and nicking, the original oligonucleotide could be amplified as either the (+)-strand or the (-)-strand. Alternatively, the hairpin structure could be removed by cleavage with the Mly I restriction enzyme, thereby releasing the oligonucleotide sequence contained within the hairpin structure from the hairpin.

**Conclusion:**

We present here a method for the enzymatic production through DNA amplification of oligonucleotides with freely designable 5'-ends and 3'-ends, using hairpin-containing self-templating oligonucleotides. The hairpin comprises recognition sequences for a nicking enzyme and a restriction enzyme. The oligonucleotides are amplified by successive rounds of ligation, rolling circle DNA synthesis and nicking. Furthermore, the hairpin can be removed by cleavage with the Mly I restriction enzyme. We have named such hairpin structures "suicide cassettes".

## Background

Single molecule detection has become an achievable goal with the development of new techniques during recent years and we have ventured into this field ourselves [1]. Some of these techniques use long chemically synthesized oligonucleotides (70–100 nucleotides) as part of the reaction set-up, e.g. padlock probes [2]. A padlock probe is a single stranded oligonucleotide which upon correct hybridization to a target sequence has its 5'-end and 3'-end brought into immediate proximity, allowing for circularization of the padlock probe by ligation. The ligation step is able to discriminate even small sequence variations in the genome [1]. These circular molecules can be detected by e.g. rolling circle DNA synthesis, where long single stranded DNA molecules comprising tandem repeats of the complementary strand of the templating circle are synthesized [3,4]. Padlock probes in combination with rolling circle DNA synthesis have been used for the in situ detection of DNA [1,5], microarray-based detection of viruses [6] and for SNP detection in combination with PCR [7,8]. Reliably achieving single molecule detection requires perfection in every step of the reaction, including perfect reagents, such as flawless oligonucleotides. A problem when using long chemically synthesized oligonucleotides is that during chemical synthesis the coupling efficiency is approximately 99% per nucleotide. For an oligonucleotide consisting of 25 nucleotides the coupling efficiency can therefore be estimated to be (0.99)^24 ^= 0.79 (24 couplings), whereas an oligonucleotide consisting of 100 nucleotides achieves an estimated coupling efficiency of (0.99)^99 ^= 0.37 (99 couplings). Furthermore, the occurrence of depurination and nucleotide skipping increases with the length of the oligonucleotide [9,10]. Thus, not only the yield but also the quality of long chemically synthesized oligonucleotides may become unsatisfactory. Some of these problems can be reduced by using one or more purification steps (e.g. PAGE and/or HPLC). However, if secondary structures are present in the oligonucleotide, high-quality purification is difficult. Numerous techniques already exist for DNA amplification of oligonucleotides through enzymatic synthesis, such as cloning [11], PCR [12] and rolling circle DNA synthesis. The latter method, the so-called circle-to-circle amplification (C2CA) [13], is a method for the amplification of a single stranded DNA sequence, based on successive rounds of ligation, rolling circle DNA synthesis and restriction digestion. Rolling circle DNA synthesis has the advantage over PCR that the multiple copies are made directly from the original circle and not as copies of copies. However, with the reliance on naturally occurring restriction sites in the oligonucleotide sequence, and on additional oligonucleotides for ligation and cleavage, C2CA is more useful for signal amplification than for production of defined oligonucleotides.

We now present a novel rolling circle method for the enzymatic production of long 5'-phosphorylated oligonucleotides with improved ligation efficiencies compared to chemically synthesized oligonucleotides and with freely designable 5'-ends and 3'-ends. This is achieved by the inclusion of a hairpin structure (termed suicide cassette) comprising binding and cleavage sites for both nicking and restriction enzymes into the starting oligonucleotide.

## Results and discussion

### Outline of the suicide cassette system

**Figure 1:**
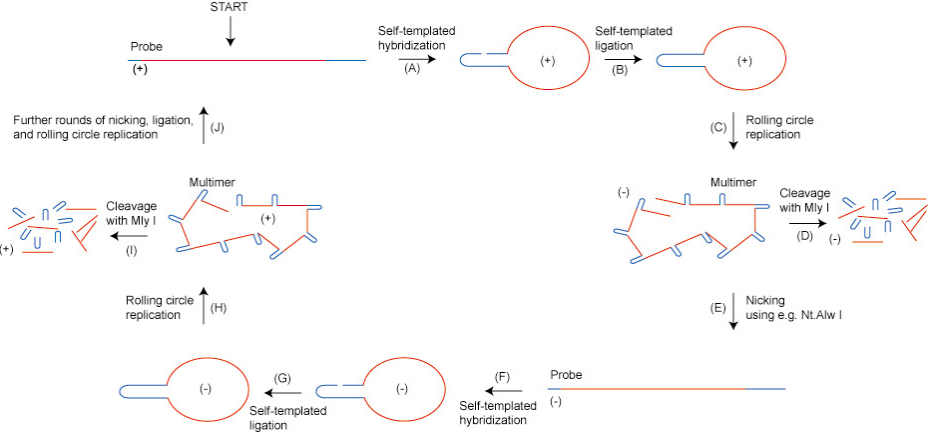
**Schematic overview of the amplification of an oligonucleotide contained within a suicide cassette**. **(A) **A DNA sequence comprising an oligonucleotide to be amplified (red) and a suicide cassette (blue) is circularized by self-templated hybridization in the suicide cassette region. **(B) **The DNA circle is closed by self-templated ligation. **(C) **By the addition of a primer, complementary to part of the DNA sequence, dNTPs and a polymerase a rolling circle DNA synthesis can be initiated resulting in tandem repeats complementary to the templating circle. **(D) **The suicide cassette can be removed from the oligonucleotide by cleavage with the Mly I restriction enzyme. **(E) **The tandem repeats can be linearized without the loss of nucleotides using the Nt. Alw I nicking enzyme, resulting in monomers complementary to the initial DNA sequence. **(F) **Self-templated hybridization (see step A). **(G) **Self-templated ligation (see step B). **(H) **Rolling circle DNA synthesis (see step C). **(I) **Cleavage with Mly I (see step D). **(J) **Nicking, using Nt. Alw I, resulting in monomers of the same polarity as the initial DNA sequence. (+) and (-) indicate the polarity of the oligonucleotide.

An oligonucleotide able to fold into a hairpin structure (the suicide cassette) by self-templated hybridization, thereby bringing the 5'-end and the 3'-end into proximity, can be circularized by the addition of a ligase [14] (Figure 1A–B). With the addition of primer, dNTP and polymerase, a rolling circle DNA synthesis can be initiated resulting in tandem repeats complementary to the circle (Figure 1C). Besides providing the template for the ligation reaction, the hairpin structure contains two additional features: I) It contains a recognition sequence for a nicking enzyme (a nicking enzyme binds double stranded DNA but cleaves only one strand of the DNA duplex). II) It contains a recognition sequence for the Mly I restriction enzyme (Mly I is, to our knowledge, the only commercially available enzyme which cleaves blunt end outside its binding sequence, as required here). Nicking allows the amplified rolling circle product to be turned into monomers without the addition of an extra oligonucleotide and without the gain or loss of nucleotides. Since the nick is positioned in the hairpin, the released monomers will have parts of the hairpin sequence positioned at each end. A denaturation-renaturation step can turn the monomers into open circle structures complementary to the original circle (Figure 1E–F). A second round of ligation and rolling circle DNA synthesis can again be followed by a nicking reaction producing oligonucleotides with the same polarity as the one purchased (Figure 1G–H and 1J). Alternatively, following each of the rolling circle steps, the products can be cleaved with Mly I, releasing the hairpin (the suicide cassette) from the rest of the oligonucleotide (Figure 1D and 1I). Although the amplification is only linear in each step of rolling circle DNA synthesis, combining rounds of amplification makes the amplification-level exponential. Since, in theory, an unlimited number of ligation, rolling circle DNA synthesis and nicking reactions could be performed, a massive amplification is possible. Unlike C2CA, the method presented does not require the additional production of oligonucleotides for both cleavage of the rolling circle products and ligation of linear products, and, equally important, the method presented provides free design of the ends of the DNA sequence to be amplified, since they are not defined by the chance presence of a binding/cleavage site for a restriction endonuclease. Instead, ends can be randomly chosen because the unique blunt end cleavage of the Mly I restriction enzyme recognizes and removes the suicide cassette added for amplification purposes.

Leaving the suicide cassette on the oligonucleotide provides a new class of circle probes, which, due to their ability to self-circularization, may have advantages for the detection of RNA (Stougaard et al., manuscript under revision).

### Detailed description of the suicide cassette

**Figure 2:**
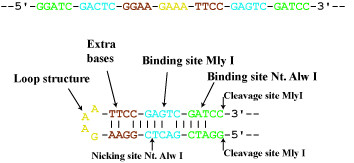
**Example of the sequence composition of a suicide cassette**. The suicide cassette contains a binding site (green) and a cleavage site (indicated by a vertical arrow) for the nicking enzyme Nt. Alw I and a binding site (blue) and a cleavage site (indicated by two vertical arrows) for the restriction endonuclease Mly I. Furthermore, the cassette comprises a loop structure (yellow) and extra bases for improving the cleavage and ligation steps (red). **Top**: Linear sequence. **Bottom**: Base pairing in a hairpin structure.

The described suicide cassette is a hairpin-structure comprising binding and cleavage sites for both the nicking enzyme Nt. Alw I and the restriction enzyme Mly I (Figure 2). During amplification only the loop of the suicide cassette will change polarity, since each strand of the stem is complementary to the opposite strand. We have used the loop structure TTTC which is replicated to GAAA following one round of amplification. GAAA constitutes a loop with a very high melting temperature probably resulting from a pseudo-base pair between the G and the last A [15,16]. Other loops such as AAAA, TTTT, TTATT and AATAA are also functional for our approach (data not shown). Four additional base pairs are positioned adjacent to the loop to improve ligation following nicking with Nt. Alw I (Figure 2).

### Amplification of an oligonucleotide contained within a suicide cassette

**Figure 3:**
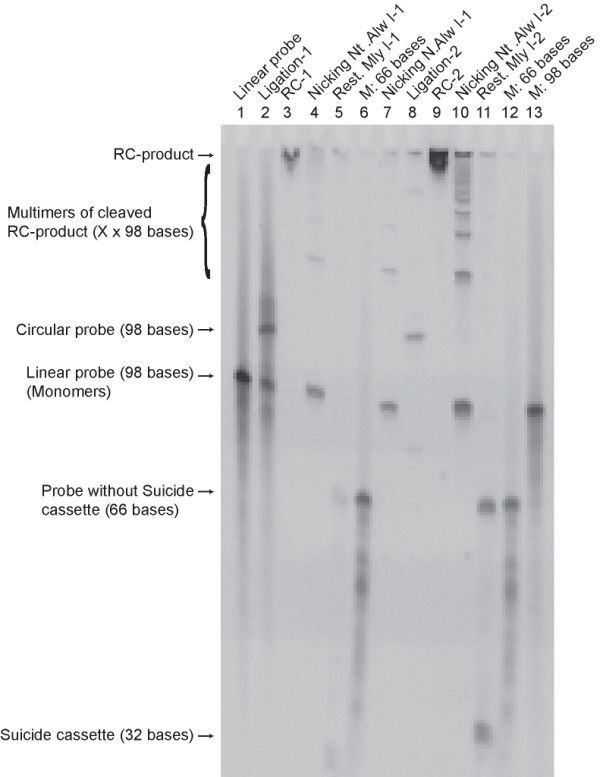
**Gel-based visualization of the oligonucleotide SF-WT90 amplified using the suicide cassette principle**. **Lane 1**: Linear probe (SF-WT90).**Lane 2**: Circularized probe ((+)-strand). Note the incomplete ligation and the background smear. **Lane 3**: Circularized probe from lane 2 amplified by rolling circle DNA synthesis. **Lane 4**: Nicked RC1-product (Nt. Alw I). **Lane 5**: Cleaved RC1-product (Mly I). **Lane 7**: Same as lane 4. **Lane 8**: Circularized probe ((-)-strand). Note the apparently complete ligation and the absence of a background smear. **Lane 9**: Circularized probe from lane 8 amplified by rolling circle DNA synthesis. **Lane 10**: Nicked RC2-product (Nt. Alw I). Lane 11: Cleaved RC2-product (Mly I).**Lane 6, 12 and 13**: Size markers. Primers used: Pr SF-WT90 (+) and Pr SF-WT90 (-). The gel was stained with SYBR Gold.

To verify the potential of our amplification technique we tried to amplify a 98-mer oligonucleotide (SF-WT90) containing a suicide cassette. Sequences with the original polarity will be called (+)-strands and sequences with the complementary polarity will be called (-)-strands. The oligonucleotide was circularized using the T4 DNA ligase, creating a product with a slower migration rate than the linear substrate when separated by denaturing PAGE (Figure 3, lanes 1–2). The rolling circle DNA synthesis created a long single stranded product consisting of tandem repeats of the complementary sequence of the original oligonucleotide, which was too long to enter the gel-matrix and could therefore only be visualized as a dot in the gel slot (Figure 3, lane 3). The rolling circle product could be cut into (-)-strand monomers without the loss of nucleotides by the nicking enzyme Nt. Alw I (Figure 3, lane 4), and the suicide cassette could be released from the rest of the oligonucleotides by Mly I (Figure 3, lanes 5–6). The nicked product could again be circularized using the T4 DNA ligase, creating circles with the (-)-polarity (Figure 3, lanes 7–8). Comparing the ligation efficiencies of the chemically synthesized oligonucleotide and the enzymatically synthesized oligonucleotide, the difference was obvious; the enzymatically synthesized oligonucleotides ligate more efficiently (compare Figure 3, lanes 1–2 and lanes 7–8). Further rounds of rolling circle DNA synthesis, nicking and ligation could be performed to get the desired amount and correct polarity of the oligonucleotides (Figure 3, lanes 9–11). The purity of the HPLC-purified oligonucleotides in lane 1 is not impressive. This is possibly due to the highly structured oligonucleotide, which is difficult to purify, since dimers can be formed during the purification step. The level of amplification (of the oligonucleotide complementary to the templating circle) after rolling circle DNA synthesis and cleavage was approximately 400 fold (estimated by PAGE, data not shown). Taken together, these results showed that successive rounds of ligation, nicking and rolling circle DNA synthesis allows for the amplification of an oligonucleotide. The nicking reactions were not complete, resulting in visible oligomers in the gel. Most likely this could be avoided by further optimization of I) enzyme:DNA ratio, II) the nicking enzyme of choice and III) optimization of the suicide cassette sequence. However, for the amplification reaction per se this phenomenon was of little significance, so we were satisfied with the results as they appear here.

### Solid support amplification to verify purity and ligation of the enzymatically synthesized oligonucleotides

**Figure 4:**
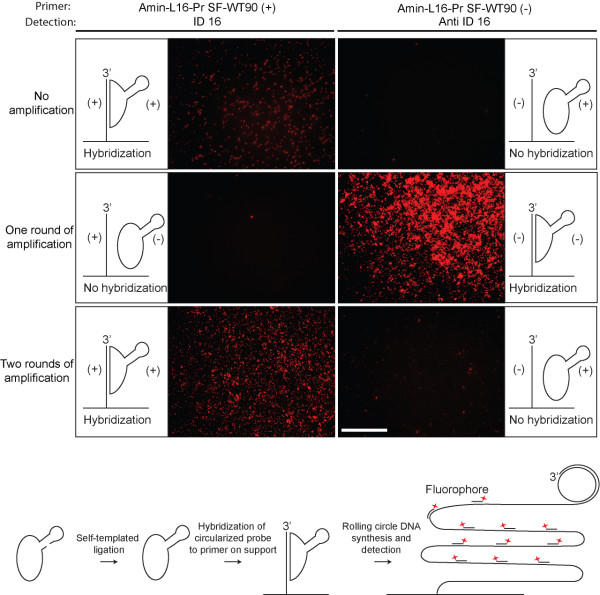
**Solid support rolling circle DNA synthesis from amplified SF-WT90 oligonucleotide nicked with Nt. Alw I**. **First row**: The chemically synthesized oligonucleotide, SF-WT90, was ligated to create a closed circle and incubated with a covalently coupled primer (Amin-L16-Pr SF-WT90 (+) or Amin-L16-Pr SF-WT90 (-)). Following a stringent wash the hybridized circles were amplified by rolling circle DNA synthesis and detected by hybridization to the rolling circle products of either the ID 16 or the anti ID 16 detection oligonucleotides. **Second row**: The SF-WT90 oligonucleotide, was amplified one round by the method presented in figure 1 (from (+)-strand to (-)-strand). Subsequently, it was ligated to create a closed circle and incubated with a covalently coupled primer (Amin-L16-Pr SF-WT90 (+) or Amin-L16-Pr SF-WT90 (-)). Following a stringent wash the hybridized circles were amplified by rolling circle DNA synthesis and detected by hybridization to the rolling circle products of either the ID 16 or the anti ID 16 detection oligonucleotides. **Third row**: The SF-WT90 oligonucleotide was amplified two rounds by the method presented in figure 1 (from (+)-strand to (-)-strand and back to (+)-strand). Subsequently, it was ligated to create a closed circle and incubated with a covalently coupled primer (Amin-L16-Pr SF-WT90 (+) primer or Amin-L16-Pr SF-WT90 (-)). Following a stringent wash the hybridized circles were amplified by rolling circle DNA synthesis and detected by hybridization to the rolling circle products of either the ID 16 or the anti ID 16 detection oligonucleotides. Schematic representations indicate the expected outcome of the hybridization events. (+) and (-) indicate the polarity of the probes. The (+)-primer hybridizes to the (+)-probe and the (-)-primer hybridizes to the (-)-probe. Equimolar amounts of probe were applied in each reaction (0.1 μM). Scale bar, 100 μm. At the bottom of the figure a schematic representation of the individual steps in the solid support assay is presented.

As we did not purify the amplification products, we wanted to test if the presence of small amounts of oligonucleotides with the opposite polarity would affect the performance of an amplified SF-WT90 probe in a hybridization assay. Primers able to hybridize to either the (+)-strand (Amin-L16-Pr SF-WT90 (+)) or the (-)-strand (Amin-L16-Pr SF-WT90 (-)) of the amplified oligonucleotide were covalently linked to a solid support. After one and two rounds of amplification the nicked products were ligated (after self-templated hybridization) and hybridized onto the solid support. As a control for hybridization and ligation an un-amplified probe was used. Solid support rolling circle DNA synthesis has previously been described by Lizardi PM [5]. Working from the amplification levels estimated by gel electrophoresis, we diluted the different generations of amplification products correspondingly in an attempt to apply equimolar amounts of probe in each reaction. Following hybridization, a rolling circle DNA synthesis was performed on the support and the products were visualized by hybridization of a labeled detection probe (ID 16 or anti ID 16) to the rolling circle products (Figure 4). All products seemed reasonably clean, in that largely no signals from circles of the opposite polarity appeared, despite the application of massive amounts of probe (0.1 μM final).

**Figure 5:**
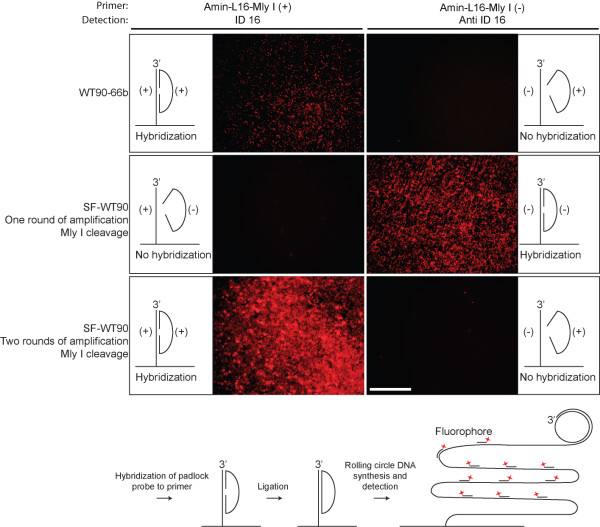
**Solid support rolling circle DNA synthesis from amplified SF-WT90 oligonucleotide cleaved with Mly I**. **First row**: The chemically synthesized padlock probe, WT90-66b, was incubated with a covalently coupled primer (Amin-L16-Mly I (+) primer or Amin-L16-Mly I (-)) in the presence of T4 DNA ligase. Only correctly hybridized padlock probes of the right sequence can be circularized. Ligated probes were subsequently amplified by rolling circle DNA synthesis and detected by hybridization to the rolling circle products of either the ID 16 or the anti ID 16 detection oligonucleotide. **Second row**: The SF-WT90 oligonucleotide, was amplified one round by the method presented in figure 1 (from (+)-strand to (-)-strand) and the suicide cassette was removed by cleavage with Mly I. Subsequently, the padlock probe generated was incubated with a covalently coupled primer (Amin-L16-Mly I (+) or Amin-L16- Mly I (-)) in the presence of T4 DNA ligase. Only correctly hybridized padlock probes of the right sequence can be circularized. Ligated probes were subsequently amplified by rolling circle DNA synthesis and detected by hybridization to the rolling circle products of either the ID 16 or the anti ID 16 detection oligonucleotide. **Third row**: The SF-WT90oligonucleotide was amplified two rounds by the method presented in figure 1 (from (+)-strand to (-)-strand and back to (+)-strand) and the suicide cassette was removed by cleavage with Mly I. Subsequently, the padlock probe generated was incubated with a covalently coupled primer (Amin-L16-Mly I (+) primer or Amin-L16- Mly I (-)) in the presence of T4 DNA ligase. Only correctly hybridized padlock probes of the right sequence can be circularized. Ligated probes were subsequently amplified by rolling circle DNA synthesis and detected by hybridization to the rolling circle products of either the ID 16 or the anti ID 16 detection oligonucleotide. Schematic representations indicate the expected outcome of the hybridization events. (+) and (-) indicate the polarity of the probes. The (+)-primer hybridizes to the (+)-probe and the (-)-primer hybridizes to the (-)-probe. Equimolar amounts of probe were applied in each reaction (0.1 μM). Scale bar, 100 μm. At the bottom of the figure is a schematic representation of the individual steps in the solid support assay.

To test for the ability to cleave the rolling circle product with Mly I, thereby creating e.g. a padlock probe, we cleaved the rolling circle products with Mly I after one and two rounds of amplification, releasing potential padlock probes from the suicide cassette. These padlock probes were hybridized to primers of both polarities (Amin-L16-Mly I (+) and Amin-L16-Mly I (-)), which were covalently linked to a solid support and then ligated (Figure 5). As a control for hybridization and ligation the padlock probe WT90-66 was used (WT90-66 is identical to the probe released from SF-WT90 following two rounds of amplification and cleavage with Mly I (from (+)-strand to (-)-strand and back to (+)-strand)). Following hybridization and ligation, the probes were amplified by rolling circle DNA synthesis and subsequently visualized by hybridization of a labeled detection probe (ID 16 or anti ID 16). This verified that padlock probes can indeed be synthesized using suicide cassettes and, as with the nicking enzyme, predominantly rolling circle products of the expected polarity were produced. Again, equimolar amounts of probe were applied in each reaction (0.1 μM final).

**Figure 6:**
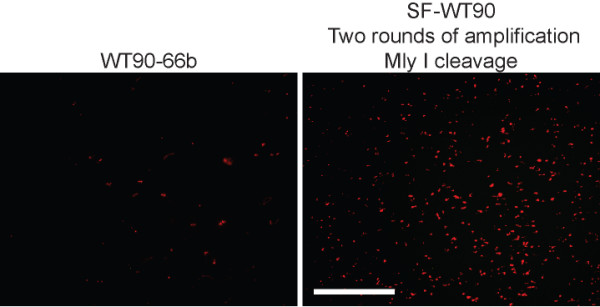
**Comparison of the chemically synthesized oligonucleotide WT90-66b and the oligonucleotide contained within the suicide cassette in SF-WT90 following amplification in a solid support rolling circle DNA synthesis assay**. The chemically synthesized padlock probe WT90-66b (left) and SF-WT90 (amplified two rounds (from (+)-strand to (-)-strand and back to (+)-strand) and cleaved with Mly I; the probe was purified by PAGE after each round) (right) were incubated with a covalently coupled primer (Amin-L16-Mly I (+)) in the presence of T4 DNA ligase. Only correctly hybridized padlock probes of the right sequence can be circularized. Ligated probes were subsequently amplified by rolling circle DNA synthesis and detected by hybridization to the rolling circle products of the ID 16 detection oligonucleotide. Equimolar amounts of probe were applied in each reaction (0.1 nM). Scale bar, 100 μm.

To test if the ligation efficiency was improved we compared the enzymatically produced oligonucleotide to a chemically synthesized oligonucleotide with the same expected sequence; this was done in a solid support assay as described in Figure 5. Additionally, the amplified probe was purified by PAGE after the first nicking reaction (from (+)-strand to (-)) and after Mly I cleavage (from (-)-strand to (+)) to minimize the background. Since the solid support assays in Figures 4 and 5 were used to test if any oligonucleotides of the wrong polarity would be amplified in a hybridization assay a high concentration of probes were applied (0.1 μM final). To be able to compare ligation efficiencies we now did a limiting dilution analysis of the probes. At a final concentration of 0.1 nM, only few signals were obtained using the chemically synthesized oligonucleotide (WT90-66b), whereas the enzymatically produced oligonucleotide still gave plenty of signals (Figure 6). This result is in agreement with a higher ligation efficiency of the enzymatically produced oligonucleotide.

## Conclusion

We present a novel method for the production of 5'-phosphorylated oligonucleotides amplifying a hairpin-containing oligonucleotide through rolling circle DNA synthesis. The hairpin is equipped with binding and cleavage sites for a nicking enzyme, which enables nicking and ligation without the addition of a supplementary oligonucleotide. Furthermore, the hairpin contains binding and cleavage sites for the Mly I restriction enzyme, enabling removal of the complete hairpin from the rest of the oligonucleotide. This method can be used for production of high quality 5'-P-oligonucleotides with superior ligation efficiencies.

## Methods

### Oligonucleotides

All oligonucleotides were purchased from DNA Technology A/S, Aarhus, Denmark and the sequences are listed in Table 1.

**Table 1:** Oligonucleotide list

**Name**	**Sequence**
WT90-66b	5'-P-CTGCCATCTT AACAAACCCT CGACCTCAAT GCTGCTGCTG TACTACTCTT ATGCGATTAC CGGGCT
SF-WT90	5'-P-GTCGATCCCT GCCATCTTAA CAAACCCTCG ACCTCAATGC TGCTGCTGTA CTACTCTTAT GCGATTACCG GGCTGGATCG ACTCGGAATT TCTTCCGA-3'
Pr SF-WT90 (+)	5'-GTAGTACAGC AGCAGCATTG AGG-3'
Pr SF-WT90 (-)	5'-CCTCAATGCT GCTGCTGTAC TAC-3'
Amin-L16-Pr SF-WT90 (+)	5'-AMIN-CCTTCCTTCC TTCCTTGTAG TACAGCAGCA GCATTGAGG-3'
Amin-L16-Pr SF-WT90 (-)	5'-AMIN-CCTTCCTTCC TTCCTTCCTC AATGCTGCTG CTGTACTAC-3'
Amin-L16-Mly I (+)	5'-AMIN-CCTTCCTTCC TTCCTTTTGT TAAGATGGCA GAGCCCGGTA ATCGCA-3'
Amin-L16-Mly I (-)	5'-AMIN-CCTTCCTTCC TTCCTTTGCG ATTACCGGGC TCTGCCATCT TAACAA-3'
ID 16	5'-TAMRA-CCTCAATGCT GCTGCTGTAC TAC-3'
Anti ID 16	5'-TAMRA-GTAGTACAGC AGCAGCATTG AGG-3'

### Amplification of DNA oligonucleotides

5'-phosphorylated oligonucleotides were ligated with T4 DNA ligase (Fermentas, Vilnius, Lithuania). Rolling circle DNA synthesis was performed for 16 hours at 37°C in a mixture containing 1× Phi29 DNA polymerase buffer (Fermentas), 1.5 nM ligated oligonucleotide, 1.5 nM primer, 250 μM dNTP, and 0.25 u/μl Phi29 DNA polymerase (Fermentas). The reactions were stopped by heat inactivation for 10 minutes at 65°C. Nicking was performed by the addition of 1 volume of a nicking mixture containing 1× NEBuffer 2 (NEB, Ipswich, MA, USA) and 1 u/μl of Nt. Alw I (NEB). Nicking reactions were incubated for 16 hours at 37°C and stopped by heat inactivation for 20 minutes at 80°C. The amount of cleavage products can be estimated by PAGE. Ligation reactions were performed by the addition of ATP and ligase to the inactivated reaction mixtures. The second round of rolling circle DNA synthesis was performed as the first one using a primer complementary to the one used in the first round. Further rounds of amplification can be performed as described above. To remove the suicide cassette, thereby terminating the amplification, cleavage of the rolling circle product was done by the addition of 1 volume of cleavage mixture containing 1× NEBuffer 4 (NEB), 0.2 μg/μl BSA (NEB) and 1 u/μl of Mly I (NEB). Cleavage reactions were incubated for 16 hours at 37°C and stopped by heat inactivation for 20 minutes at 65°C. Products can be separated and purified by PAGE.

### Polyacrylamid gel electrophoresis (PAGE)

Amplified and cleaved products could be separated by PAGE. Products were separated on 10% denaturating gels and stained with SYBR Gold. Images were visualized using a Gel Doc 1000 (Biorad).

When indicated the cleaved products were purified from the gel using standard phenol/chloroform purification and the concentration estimated by photospectrometry.

### Solid support amplification and detection of Nt. Alw I and Mly I cleaved rolling circle products

5'-amine coupled primers were linked to CodeLink Activated Slides (GE Healthcare) according to the manufacturers protocol. *Detection after nicking*: Nicked products (actual concentrations in individual experiments are given in the figure legends) were ligated in 1× ligation buffer (Fermentas), inactivated, supplemented with 0.5 M NaCl (final concentration) and hybridized to the solid support for 30 minutes at 37°C in a humidity chamber. *Detection after Mly I cleavage*: Cleaved products (actual concentrations in individual experiments are given in the figure legends) were ligated directly onto the coupled primes in a mixture containing 1× ligase buffer (Fermentas), 0.2 μg/μl BSA, 250 mM NaCl, and 0.05 u/μl T4 DNA ligase (Fermentas) for 30 minutes at 37°C in a humidity chamber. The slides were washed in 0.1 M Tris-HCL, 150 mM NaCl and 0.3% SDS (wash buffer 1) for 2 min at room temperature followed by a wash in 0.1 M Tris-HCL, 150 mM NaCl and 0.05% Tween-20 (wash buffer 2). Rolling circle DNA synthesis was performed for 30 minutes at 37°C in 1× Phi29 buffer, 0.2 μg/μl BSA, 250 μM dNTP, 5% glycerol, and 1u/μl Phi29 DNA polymerase. The reactions were terminated by washing 2 minutes in wash buffer 1 and 2 minutes in wash buffer 2. The rolling circle products were detected by hybridizing fluorescently labeled oligonucleotides in a buffer containing 20% formamide, 2× SSC, 5% glycerol and 0.17 μM of either ID 16 or anti ID 16 for 30 min at 37°C. Slides were washed for 2 min at room temperature in wash buffer 1 and wash buffer 2 and dehydrated through a series of ethanol and mounted with Vectashield (Vector Laboratories, Burlingame, CA, USA).

### Image analysis

Solid support assays were analyzed with an epifluorescence microscope (Leica, Wetzlar, Germany) and images were recorded with a SenSys CCD-camera operated by the SmartCapture 2 version 2.0 from Digitalscientific (Cambridge, UK). A 63× objective (Leica) was used for all images. Thresholding was performed using Adobe Photoshop (Adobe Systems).

## Authors' contributions

JSL contributed to the conception of the method, probe design, performed all experiments in the laboratory, and drafted the manuscript. MS participated in method development, probe design, data analysis and revision of the manuscript. JK initiated the project and contributed with data analysis and critical revision of the manuscript. All authors read and approved the final manuscript.
